# Damaged-DNA Binding Protein-2 Drives Apoptosis Following DNA Damage

**DOI:** 10.1186/1747-1028-5-3

**Published:** 2010-01-19

**Authors:** Srilata Bagchi, Pradip Raychaudhuri

**Affiliations:** 1 Center of Molecular Biology of Oral Diseases (M/C 860), College of Dentistry, Cancer Center, University of Illinois at Chicago, 801 S. Paulina Ave, Chicago, IL-60612, USA; 2 Department of Biochemistry and Molecular Genetics (M/C 669), Cancer Center, University of Illinois at Chicago, 900 S. Ashland Ave, Chicago, IL-60607, USA

## Abstract

Apoptosis induced by DNA damage is an important mechanism of tumor suppression and it is significant also in cancer chemotherapy. Mammalian cells activate the pathways of p53 to induce apoptosis of cells harboring irreparable DNA damages. While p53 induces expression of various pro-apoptotic genes and directly participates in the disruption of mitochondrial membrane polarization, it also increases expression of the cell cycle inhibitor p21 that is a dominant inhibitor of caspase-activation and apoptosis. Here we discuss how Damaged-DNA Binding Protein-2 (DDB2) subdues the level of p21 in cells harboring irreparable DNA damage to support activation of the caspases. We speculate a model in which DDB2 detects and couples the presence of un-repaired DNA damages to the proteolysis of p21, leading to the induction of apoptosis.

## Review

### Cell cycle inhibitor p21 inhibits apoptosis following DNA damage

The DNA damage response pathways are critical mechanisms of tumor suppression, as these pathways prevent accumulation of cells with oncogenic mutations. Often the DNA repair pathways are insufficient to repair all the damages. Under these circumstances permanent arrest (premature senescence) or apoptosis is induced to prevent accumulation of cells with oncogenic mutations. It is unclear how cells sense the presence of irreparable DNA damage - a situation when the extent of damage is greater than repair-capacity of a cell. It is expected that premature senescence or apoptosis would be induced only after the cell has a window of opportunity to repair the damages. A likely scenario is that a continued activation of the ATM/ATR beyond the attempts to repair the damages is responsible for the induction of premature senescence or apoptosis. Apoptosis is clearly the most effective tumor suppression mechanism following DNA damage because it eliminates the cells harboring irreparable DNA damages. The tumor suppressor p53 is considered to be the central activator of the DNA damage-induced apoptosis. Activated ATM/ATR cause stabilization and activation of p53 (Reviewed in [1]). Once activated, p53 directly and indirectly causes disruption of mitochondrial membrane polarization, leading to the activation of caspases - Apoptosis ensues.

Mechanisms of p53 involve transcriptional activation of pro-apoptotic genes and inhibition of anti-apoptotic genes. For example, p53 transcriptionally activates expression of PUMA, BID, BAX, and NOXA (Reviewed in [2]) to induce apoptosis. Also, the anti-apoptotic protein Survivin is inhibited by p53 [3]. In addition to these mechanisms, p53 also translocates to mitochondria and directly binds to Bcl-2 and inhibits its anti-apoptotic activity. This latter phenomenon is termed transcription-independent p53-induced apoptosis or TIPA [4]. While p53 activates the pro-apoptotic pathways, the p53-induced cell cycle-inhibitory protein p21 was shown also to be a potent inhibitor of apoptosis [5-8]. Expression of p21 is induced also by E2F1, which is another pro-apoptotic gene [9,10]. In addition to stabilizing p53, the ATM/ATR pathways stabilize E2F1, which induces apoptosis through both p53 and p53-independent pathways [[10] and references therein].

The p53-mediated induction of p21 is partly responsible for the cell cycle delays observed after DNA damage that is accomplished through inhibition of CDKs. The CDK-inhibitory function of p21 is linked also to its anti-apoptotic activity [6], and it has been suggested that the execution of apoptosis requires CDK-activity [11]. However, the mechanisms by which CDKs participate in apoptosis or p21 inhibits activation of the caspases are unclear. Nevertheless, the increase in p21 following DNA damage is important, as it inhibits the apoptotic pathway to allow a window of opportunity for cells to repair the damaged-DNA. (However, a very high-level of p21, as seen in the DDB2-/- cells, is inhibitory to repair [[12] and references therein]) Thus, the increased expression of p21 after DNA damage can be viewed as a survival pathway. For cells to undergo apoptosis after DNA damage, mechanisms must exist that reduce the level of p21 to support activation of the caspases. Theoretically, a continued activation of ATM/ATR by the un-repaired DNA damages will only increase the level of p21 through stabilization of p53 and E2F1. On the other hand, extinguishing the checkpoint pathways of ATM and ATR is expected to lower the level of p21. Alternatively, a pathway that overrides the p53-p21 inhibitory loop without affecting the pro-apoptotic function of p53 might be significant in driving apoptosis following DNA damage. The xeroderma pigmentosum group E (XP-E) gene product DDB2 fits that bill nicely.

DDB2 is a substrate-adapter for the E3 ubiquitin ligase Cul4-DDB1 [13]. In human cells, but not in rodent cells, expression of DDB2 is induced by p53 [14]. Like p21, expression of DDB2 in human cells also increases after DNA damage [14]. Recent studies by Stoyanova et al. [15] demonstrated that DDB2 targets p21 for ubiquitination and proteolysis in cells harboring DNA damage. Under these conditions, DDB2 has no effect on the pro-apoptotic pathway of p53 [15]. Below, we discuss how DDB2 mediated proteolysis of p21 is important for cells to undergo apoptosis following exposure to DNA damaging agents.

### Targeting p21 for proteolysis after DNA damage

P21 is targeted for proteasome-mediated proteolysis by an impressive number of pathways: Both ubiquitin-dependent and -independent pathways have been shown to degrade p21. One study indicated that mutations of all lysine-residues, which inhibited ubiquitination, had no effect on the stability of p21 [16]. Also, the N-terminus of p21 was found to be acetylated, suggesting that p21 is degraded by a ubiquitin-independent mechanism. It was shown that the ubiquitin-independent mechanism involved the REGgamma complex [16,17]. Another study indicated a role of Mdm2 in targeting p21 for proteolysis by a ubiquitin-independent mechanism [18]. Other studies, on the other hand, demonstrated ubiquitin-dependent proteolysis of p21. First, several reports indicated an involvement of the SCF-SKP2 ligase for the ubquitination and proteolysis of p21 in late G1 and in S phases [19-22]. At G2/M phase, however, p21 was shown to be poly-ubiquitinated by the APC/C-Cdc20 ligase complex [23]. In addition to the SCF and APC/C ligases, the Cul4-DDB1 ligase has been shown to target p21 for proteolysis. Interestingly, targeting by the Cul4-DDB1 ligase involves PCNA and the substrate adapter Cdt2 [24,25]. This pathway has been implicated in the proteolysis of p21 also in the UV-irradiated cells. A PCNA-binding mutant of p21 was not degraded after UV irradiation. Also, a phosphomimetic substitution at Ser114 of p21, a site that is phosphorylated by ATR after UV irradiation [26], enhanced polyubiquitination by the Cul4-DDB1-Cdt2 ligase [24]. On the other hand, another study reported evidence for an unidentified ubiquitin-independent mechanism for the proteolysis of p21 phosphorylated at Ser114 after UV irradiation [27]. Although many of these studies reported their results in a controversial manner, it is obvious that the mammalian cells have evolved to utilize multiple pathways to insure efficient degradation of p21 in dividing cells to allow unperturbed progression through the phases of the cell cycle. Therefore, the inhibitory function of p21 is restricted to specific physiological context (such as exposure to DNA damaging agents) in which the rate of synthesis is greater than rate of degradation.

Studies on the DDB2-/- MEFs resulted in the identification of yet another mechanism for ubiquitin-dependent proteolysis of p21 [15]. Interestingly, DDB2-/- MEFs exhibited only a marginal increase in the level of p21. By contrast, following treatments with DNA damaging agents, the DDB2-/- MEFs accumulated p21 at a much higher level compared to the wild-type MEFs. The high-level accumulation of p21 did not result from a relatively greater increase in expression. Rather there was a significant increase in the half-life of the p21 protein in the DDB2-/- MEFs. DDB2-deficient human cells also exhibited a similar increase in the stability of p21 following treatment with DNA damaging agents. Moreover, the DDB2-deficient human cells exhibited a reduction in the polyubiquitination of p21 compared to the DDB2-proficient cells, indicating that the DDB2-mediated proteolysis of p21 following DNA damage involves polyubiquitination of p21. DDB2 could bind p21, and the binding ability was increased in cells harboring DNA damage [15]. These observations indicated a significant role of DDB2 in the proteolysis of p21 following DNA damage.

The reduced polyubiquitination and proteolysis of p21 in the DDB2-deficient cells following DNA damage are somewhat surprising given that p21 can be degraded by multiple ubiquitin-dependent and -independent pathways. Nevertheless, the high-level accumulation of p21 suggests that the DDB2-mediated proteolysis of p21 is critical following DNA damage. The DDB2-binding partner DDB1 has been implicated in the proteolysis of p21 [24,28]. Moreover, DDB2 in conjunction with DDB1 associates with Cul4 to form a functional E3 ligase. Therefore, it is possible that a complex of Cul4, DDB1 and DDB2 is involved in the ubiquitination and proteolysis of p21 after DNA damage. On the other hand, since the Cul4-DDB1-Cdt2 complex has been implicated in the proteolysis of p21 after UV irradiation [24,25], it is possible that DDB2 collaborates with that complex to target p21 for proteolysis. The possibility that DDB2 is required for multiple pathways of p21-proteolysis could not be ruled out. For example, DDB2 increases nuclear level of DDB1 following UV irradiation [12,29], causing an increase in the nuclear abundance of the Cul4-DDB1 ligase. But, the interaction between DDB2 and p21, and its increase following DNA damages suggest a more direct role of DDB2 in targeting p21 for ubiquitination. In that regard, it will be important to consider the possibility that DDB2 plays a role in localizing p21 to the damaged-chromatin for proteolysis by an available E3 ubiquitin ligase (such as the Cul4-DDB1-Cdt2 complex).

### DDB2-mediated proteolysis of p21 is a key activator of apoptosis following DNA damage

Linn laboratory demonstrated that the DDB2 -/- MEFs as well as XP-E cells are deficient in apoptosis following UV irradiation [30,31]. These results were confirmed by Stoyanova et al. [15]. Moreover, Stoyanova et al. further demonstrated that the DDB2-deficienct cells are impaired in apoptosis induced by ionizing radiation, cisplatin and aclarubicin treatment. Furthermore, E2F1-induced apoptosis also involved DDB2 [15]. The deficiencies in apoptosis following DNA damage was observed also in lymphocytes and in keratinocytes isolated from the DDB2 -/- mice (unpublished observations of Stoyanova and Raychaudhuri). The deficiency in apoptosis did not result from a lack of expression of the p53-induced pro-apoptotic genes. PUMA and BAX were efficiently induced in the DDB2-/- MEFs following treatments with the DNA damaging agents. Moreover, cell fractionation studies indicated that the DNA damage-induced localization of p53 to mitochondria was not affected in the DDB2-deficient cells. However, the activation of caspase 3 and PARP cleavage were impaired, suggesting that the DDB2-deficient cells are impaired in caspase activation following disruption of mitochondrial membrane polarization.

CDK-inhibitors were shown to strongly inhibit ionizing radiation-induced caspase processing and activation without affecting the mitochondrial events [6]. It has been suggested that the activation of caspases requires CDK-activity. Interestingly, in the DDB2-/- MEFs, the lack of DNA damage-induced apoptosis coincided with the high-level accumulation of p21 [15]. Moreover, those cells exhibited a greatly reduced CDK-activity. To determine whether the accumulation of p21 was responsible for the blockade of apoptosis in the DDB2-deficiencient cells, Stoyanova et al. deleted p21 in the DDB2-/- background. The DDB2-/-p21-/- MEFs did not exhibit any deficiency in apoptosis following DNA damage. Moreover, the E2F1-induced apoptosis also was restored in the DDB2-/-p21-/- MEFs. Together those observations clearly demonstrated that the lack of proteolysis of p21 and consequent accumulation of p21 in the DDB2-deficient cells is responsible for the inhibition of apoptosis following DNA damage.

A recent study indicated that Mdm2, along with p21, is required for efficient inhibition of CDK2 in cells treated with DNA damaging agents [32]. SiRNA-mediated depletion of Mdm2 leads to inefficient arrest by the DNA damaging agents. In agrement with that, Stoyanova et al. showed that depletion of Mdm2 in the DDB2-deficient cells increased CDK2-activity, which was retained to a significant extent after treatment with DNA damaging agents [15]. Moreover, depletion of Mdm2 in the DDB2-deficient cells also restored apoptosis induced by DNA damaging agents [15]. These observations further support the notion that DDB2 activates CDK2 by inducing proteolysis of p21 to then allow cells to activate the caspases and undergo apoptosis following DNA damage.

### Why DDB2?

DDB2 is unique among the other E3 ligase-associated proteins that have been implicated in the proteolysis of p21 in that it has a very high affinity for damaged-DNA. It binds to UV-damaged DNA, cisplatin-modified DNA, as well as single-stranded DNA with high affinities [33]. It binds damaged-DNA in conjunction with DDB1 [[33] and references therein]. Moreover, the DDB2-DDB1 complex can also recruit Cul4 to the damaged-DNA [12,34]. The damaged-DNA binding activity of DDB2 has been implicated in its nucleotide excision repair (NER) function. It was shown that Cul4-DDB1-DDB2 complex could ubiqutinate histones [35,36]. Based on that observation, it was suggested that DDB2 participates in NER by modifying chromatin structure at the site of UV-damage. While the model is intriguing, a clear evidence for DDB2-mediated histone-ubiquitination in NER is still lacking. We found the repair-deficiencies of the DDB2-/- MEFs could be reversed by the deletion of p21 [12]. We proposed that DDB2 participates in NER indirectly by regulating the levels of p21. However, it remains possible that two mechanisms are linked: Histone-ubiquitination might be involved in the proteolysis of p21 on the damaged-chromatin.

The damaged-DNA binding activity of DDB2 might be related also to its apoptotic activity. For example, we speculate that, in addition to a potential role in repair, the damaged-DNA binding activity of DDB2 might be significant in sensing the un-repaired DNA damages when the extent of damage is greater than repair-capability of a cell. After sensing the un-repaired DNA, DDB2 channels cells to proceed through the apoptotic pathway by inducing proteolysis of the apoptosis-regulator p21. The Cul4-DDB1-Cdt2 complex was shown to induce proteolysis of Cdt1 in the context of chromatin [37]. It is possible that a similar mechanism involving damaged-chromatin is required for the proteolysis of p21 by DDB2 - Such a mechanism would couple the presence of un-repaired damaged-chromatin to the proteolysis of p21, thereby allowing apoptosis to proceed when it is important.

## Conclusion

DNA damage-induced apoptosis is significant not only as a mechanism of tumor suppression it is also a mechanism that is used by the chemotherapy drugs to eliminate cancer cells. Many of the chemotherapy drugs are also DNA-damaging agents, and they function by inducing apoptosis. Stoyanova et al. demonstrated that both normal and cancer cells lacking DDB2 are resistant to apoptosis induced by the chemotherapy drugs cisplatin and aclarubicin [15]. Thus, DDB2 is important for the therapeutic efficacies of the drugs. In support of that notion, a recent reported that expression of DDB2 sensitizes cancer cells to therapy [38]. A corollary to these observations is that reduced expression of DDB2 would lead to drug resistance of cancer cells. In that regard, it is noteworthy that DDB2 is a p53-induced gene, and p53 is mutated in greater than 50% of cancers. Consistent with that, a search of the Oncomine database revealed that DDB2 is one of the top 10% under-expressed genes in a variety of cancers (Table 1). It will be important to analyze the mechanisms, other than p53, that stimulate expression of DDB2 because the information can be used to increase expression of DDB2 in tumors and sensitize them to chemotherapeutic drugs.

**Table 1:** Reduced expression of DDB2 in cancer (Source: Oncomine)

DDB2 among top 5-10% under-expressed genes (Oncomine - Expression Signature): Cancer types	Database	References
Brain and CNS cancer	Sun Brain	Cancer Cell (2006), 9: 287-300

Colon Adenocarcinoma	Kaiser Colon	Genome Biol (2007) 8: R131

Ductal breast carcinoma	Richradson breast 2	Cancer Cell (2006) 9: 121-132

Ductal breast carcinoma	Huang Breast	Lancet (2003) 361: 1590-1596

Follicular Lymphoma	Storz	J. Invest Dermatol. (2003) 120: 865-870

T-cell Prolymph.leukemia	Durig leukemia	Leukemia (2007) 10: 2153-2163

Chronic Lymph. leukemia	Rosenwald leukemia	N Engl J Med (2002) 346: 1937-1947

Burkitt's lymphoma	Basso lymphoma	Nat. Genet. (2005) 37: 382-390

Liver cancer (HCC)	Warmbach Liver	Hepatology (2007) 45: 938-947

Liver cancer (HCC)	Chen Liver	Mol. Biol. Cell. (2002) 13: 1929-1939

FAB subtype M2	Wouter's leukemia	Blood (2009) 113: 3088-3091

Head and Neck SCC	Chung SCC	Cancer Cell (2004) 5: 489-500

Lung adenocarcinoma	Beer Lung	Nat. Med. (2002) 8:816-824

Ovarian adenocarcinoma	Welsh Ovary	PNAS (2001) 98: 1176-1181

Prostate adenocarcinoma	Vanaja Prostate	Cancer Res. (2003) 63: 3877-3882.

## Competing interests

The authors declare that they have no competing interests.

## Authors' contributions

SB and PR wrote the review.
